# Supplementary material for: Targeting oncogenic mutations in colorectal cancer using cryptotanshinone
Source: PLoS One. 2021 Feb 17;16(2):e0247190. doi: 10.1371/journal.pone.0247190 (PMC7888617; doi:10.1371/journal.pone.0247190)
Supplement: S1 Table — Ratio of probabilities (ROP) of different genes in the colorectal cancer pathway. (PDF) [file pone.0247190.s001.pdf]

Additional File 1: Ratio of probabilities (ROP) of different genes in the colorectal cancer pathway is tabulated below.

Gene Symbol	Ratio of Probabilities
AKT1	2.250
APC	1.25
BAD	0.265
BCL2	38.000
CCND1	40.000
CTNNB1	7.000
DVL2	1.769
EGF	0.850
EGFR	1.450
ELK1	3.444
ELK4	32.000
ERBB2	0.538
ERBB3	0.632
FOS	19.500
GRB2	0.526
GSK3	0.704
HBEGF	11.000
IGF1	0.340
IGF1R	2.143
IRS1	5.571
JAK1	2.143
JUN	36.000
KRAS	17.500
LEF1	7.333
MAP2K1 (MEK1)	1.286
MAP2K4 (MKK4)	2.500
MAP3K1 (MEKK1)	2.067

MAPK8 (JNK1)	5.667
MAPK1 (ERK1)	8.750
MTOR	1.688
MYCN	3.143
NRG1	0.633
PDPK1	1.444
PIK3CA	1.474
PTEN	0.240
RAC1	0.778
RAF1	5.400
RHEB	4.833
RPS6KB1	5.333
SMAD3	0.897
SOS	0.640
SP1	2.385
SRF	2.118
STAT3	6.400
TCF	3.714
TGFB1	3.286
TRAF1	1.611
MAP3K7 (TAK1)	0.966
TSC1	0.571
WNT16	2.077
