# Supplementary material for: Targeting oncogenic mutations in colorectal cancer using cryptotanshinone
Source: PLoS One. 2021 Feb 17;16(2):e0247190. doi: 10.1371/journal.pone.0247190 (PMC7888617; doi:10.1371/journal.pone.0247190)
Supplement: S2 Table — Normalized average size difference values for different drug combinations with no therapy (untreated) as the reference. (PDF) [file pone.0247190.s002.pdf]

Additional File 2: Normalized average size difference values for different drug combinations with no therapy (untreated) as the reference.

Drug Combinations	Average Size Difference Measure
Untreated	1.000
U0126	0.433
NT157	0.990
NT157 + U0126	0.424
AG1024	0.914
AG1024 + U0126	0.410
AG1024 + NT157	0.904
AG1024 + NT157 + U0126	0.400
H03867	0.875
HO3867 + U0126	0.343
HO3867 + NT157	0.864
HO3867 + NT157 + U0126	0.333
HO3867 + AG1024	0.791
HO3867 + AG1024 + U0126	0.319
HO3867 + AG1024 + NT157	0.779
Lapatinib	0.680
Lapatinib + U0126	0.331
Lapatinib + NT157	0.669
Lapatinib + NT157 + U0126	0.320
Lapatinib + AG1024	0.575
Lapatinib + AG1024 + U0126	0.302
Lapatinib + AG1024 + NT157	0.562
Lapatinib + HO3867	0.602
Lapatinib + HO3867 + U0126	0.264
Lapatinib + HO3867 + NT157	0.588
Lapatinib + HO3867 + AG1024	0.497
Temsirolimus	0.994
Temsirolimus + U0126	0.426
Temsirolimus + NT157	0.984

Temsirolimus + NT157 + U0126	0.417
Temsirolimus + AG1024	0.908
Temsirolimus + AG1024 + U0126	0.402
Temsirolimus + AG1024 + NT157	0.897
Temsirolimus + HO3867	0.868
Temsirolimus + HO3867 + U0126	0.336
Temsirolimus + HO3867 + NT157	0.857
Temsirolimus + HO3867 + AG1024	0.783
Temsirolimus + Lapatinib	0.673
Temsirolimus + Lapatinib + U0126	0.323
Temsirolimus + Lapatinib + NT157	0.661
Temsirolimus + Lapatinib + AG1024	0.567
Temsirolimus + Lapatinib + HO3867	0.594
LY294002	0.779
LY294002 + U0126	0.195
LY294002 + NT157	0.779
LY294002 + NT157 + U0126	0.195
LY294002 + AG1024	0.710
LY294002 + AG1024 + U0126	0.190
LY294002 + AG1024 + NT157	0.710
LY294002 + HO3867	0.700
LY294002 + HO3867 + U0126	0.175
LY294002 + HO3867 + NT157	0.700
LY294002 + HO3867 + AG1024	0.635
LY294002 + Lapatinib	0.519
LY294002 + Lapatinib + U0126	0.169
LY294002 + Lapatinib + NT157	0.519
LY294002 + Lapatinib + AG1024	0.435
LY294002 + Lapatinib + HO3867	0.490
LY294002 + Temsirolimus	0.770
LY294002 + Temsirolimus + U0126	0.172
LY294002 + Temsirolimus + NT157	0.770

LY294002 + Temsirolimus + AG1024	0.700
LY294002 + Temsirolimus + HO3867	0.693
LY294002 + Temsirolimus + Lapatinib	0.509
Cryptotanshinone	0.274
Cryptotanshinone + U0126	0.274
Cryptotanshinone + NT157	0.264
Cryptotanshinone + NT157 + U0126	0.264
Cryptotanshinone + AG1024	0.252
Cryptotanshinone + AG1024 + U0126	0.252
Cryptotanshinone + AG1024 + NT157	0.241
Cryptotanshinone + HO3867	0.274
Cryptotanshinone + HO3867 + U0126	0.274
Cryptotanshinone + HO3867 + NT157	0.264
Cryptotanshinone + HO3867 + AG1024	0.252
Cryptotanshinone + Lapatinib	0.202
Cryptotanshinone + Lapatinib + U0126	0.202
Cryptotanshinone + Lapatinib + NT157	0.190
Cryptotanshinone + Lapatinib + AG1024	0.175
Cryptotanshinone + Lapatinib + HO3867	0.202
Cryptotanshinone + Temsirolimus	0.266
Cryptotanshinone + Temsirolimus + U0126	0.266
Cryptotanshinone + Temsirolimus + NT157	0.255
Cryptotanshinone + Temsirolimus + AG1024	0.243
Cryptotanshinone + Temsirolimus + HO3867	0.266
Cryptotanshinone + Temsirolimus + Lapatinib	0.193
Cryptotanshinone + LY294002	0.107
Cryptotanshinone + LY294002 + U0126	0.107
Cryptotanshinone + LY294002 + NT157	0.107
Cryptotanshinone + LY294002 + AG1024	0.103
Cryptotanshinone + LY294002 + HO3867	0.107
Cryptotanshinone + LY294002 + Lapatinib	0.096
Cryptotanshinone + LY294002 + Temsirolimus	0.087
